# A gene-specific RNA enrichment protocol for nanopore direct-RNA sequencing

**DOI:** 10.1371/journal.pone.0339960

**Published:** 2026-02-11

**Authors:** Maja Bele Dyrendalsli, Cecilie Løkke, Christer Einvik

**Affiliations:** 1 Department of Pediatrics, Division of Child and Adolescent Health, UNN-University Hospital of North-Norway, Tromsø, Norway; 2 Research Group for Child and Adolescent Health, Department of Clinical Medicine, Faculty of Health Sciences, UiT-The Arctic University of Norway, Tromsø, Norway; Boyce Thompson Institute, UNITED STATES OF AMERICA

## Abstract

Oxford Nanopore direct-RNA sequencing, a third-generation sequencing technology, allows for the analysis of native RNA molecules in their natural cellular state. However, cellular RNA is predominantly composed of ribosomal RNA and transcripts from ubiquitously expressed housekeeping genes, which limits the coverage of transcripts from lowly expressed genes. To address this limitation, targeted sequencing can be employed to enrich read coverage by focusing specifically on genes of interest. Here, we present a step-by-step protocol for gene-specific RNA enrichment followed by Oxford Nanopore direct-RNA sequencing. The enrichment protocol utilizes biotinylated DNA capture probes complementary to the target gene. Following in-solution hybridization of probes to total RNA, a series of stringent washes is applied before elution of the enriched RNA sample. The protocol describes all steps from isolation of cellular total RNA to bioinformatic analyses of raw sequencing data. As a proof of concept, capture probes were designed to specifically enrich all RNA species encoded by the MYCN oncogene. The enrichment protocol successfully isolated RNAs from the MYCN gene, achieving a purification factor of 4.8 × 10^3^. Direct-RNA sequencing of the enriched RNA sample revealed that 65% of the primary mapped reads aligned to MYCN transcripts. We also include a more thorough analysis of the most abundant non-target mapped reads and unmapped reads. This protocol proves to be highly effective in removing unwanted RNA species, delivering robust enrichment for the target gene, and significantly enhancing the efficiency of long-read direct-RNA sequencing.

## Introduction

Long-read direct-RNA sequencing by Oxford Nanopore Technologies (ONT) is a powerful tool for studying full-length native RNA molecules as they exist in cells [1]. Since this technology does not rely on cDNA synthesis, PCR amplification or fragmentation steps, it preserves information of nucleotide modifications and poly(A) tail lengths and offers an advantage to accurately measure both gene and isoform expression levels in an unbiased manner [2].

Nanopore direct-RNA sequencing requires three short library preparation steps before the RNA is loaded onto a flow cell. Loading a total RNA library onto a Nanopore flow cell results in the majority of nanopores being occupied by ribosomal RNA, which accounts for approximately 80% of cellular RNA [3]. While ribodepletion or poly(A) selection can partially address this issue, most pores will still be occupied by transcripts from thousands of ubiquitously expressed genes. As many as 8000 protein-coding genes, accounting for around 75% of all mRNAs, are reported to be ubiquitously expressed [4]. These genes are typically involved in fundamental cellular processes like metabolism, transcription, RNA processing or translation.

To perform Nanopore direct RNA sequencing on focused RNA targets, either for cost effectiveness or to improve sensitivity, a protocol for selective enrichment of specific transcripts of interest is necessary. Several protocols for targeted RNA sequencing exist for technologies requiring cDNA synthesis and PCR amplification [5–7], while established methods to perform targeted direct-RNA sequencing on Nanopore platforms are lacking.

Here, we developed a gene-specific enrichment protocol tailored for Nanopore direct-RNA sequencing. The method is based on the use of DNA capture probes complementary to the RNA of interest. We designed the capture probes to target the complete *MYCN* oncogene, aiming to sequence all RNA species generated from the gene.

This RNA capture approach is versatile and can be applied to other areas of RNA research as well.

## Materials and methods

The protocol associated with this peer-reviewed article is available on protocols.io (dx.doi.org/10.17504/protocols.io.8epv52m16v1b/v1) and is also provided as S1 File for printing. This method of gene enrichment utilizes the strong bond between streptavidin and biotin, which is one of the strongest non-covalent interactions in nature [8].

In short, the target RNA was captured using specific biotinylated deoxyoligonucleotide probes that bind to streptavidin-coated magnetic beads. Details of the capture probes are provided as S1 Table. A magnet was employed to immobilize the target-bound complexes, facilitating the removal of the supernatant. Following several stringent bead washing steps, the purified sample was eluted prior to construction of the sequencing library. RT-qPCR was utilized to quantify the target gene in samples before and after the enrichment, as detailed in S1 File section 22 – *Validation of gene enrichment*. The Oxford Nanopore direct-RNA sequencing kits require the presence of a 3’ polyadenylated (poly(A)) tail on template molecules for successful library generation. To include non-polyadenylated RNAs from the enriched sample, an optional poly(A)-tailing step can be introduced, followed by a cleanup.

## Results

*MYCN* is a proto-oncogene and a member of the MYC family of transcription factors. MYC proteins regulate genes involved in proliferation, growth, senescence, metabolism, differentiation, and apoptosis [9,10]. *MYCN* plays a central role in neuroblastoma (NB), a pediatric cancer arising from neural crest cells that typically affects the adrenal glands and sympathetic nervous system [11]. *MYCN* amplification is strongly linked to poor prognosis and treatment failure among NB patients [12,13]. *MYCN* is highly expressed in *MYCN*-amplified (MNA) NB cell lines, like Kelly cells, making it a good candidate for the development of this protocol.

**Fig 1:**
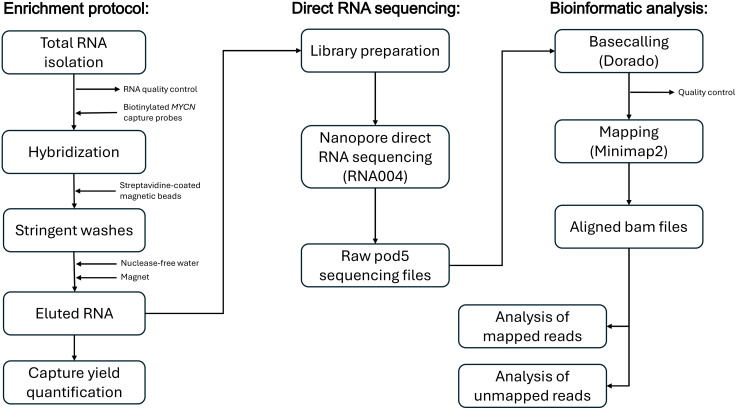
Method flow chart. Overview of the workflow for the enrichment protocol followed by direct RNA sequencing and bioinformatic analyses.

We performed the gene-specific RNA enrichment protocol using a 60-nt uniform 2X tilling pool of *MYCN* capture probes followed by direct-RNA sequencing as described in S1 File. The entire workflow, from total RNA to bioinformatic analyses, is illustrated by a method flow chart in Fig 1. The *MYCN* capture probe pool was designed to cover the complete *MYCN* gene sequence, including both introns.

### Capture yield quantification

To quantify the purification of *MYCN* RNA after enrichment, RT-qPCR was used to compare the pre- and post-capture RNA libraries. The beta-actin (*ACTB*) mRNA was used as an endogenous control. The purification factor was calculated using the relationship between *MYCN* and *ACTB* pre- and post-capture (Table 1, S1 File section 22).

**Table 1:** Calculations of the purification factor. Pre-capture (10 ng total RNA before enrichment) and post-capture (3 µl enriched RNA) samples were quantified using RT-qPCR. The second and third columns show N0 values (italic) and relations. N0 is the qPCR efficiency-corrected target quantity as reported by LinRegPCR (v.2021.2). The final purification factor is shown in bold.

	pre-capture	post-capture
*MYCN*	3.96*10 ^*−7*^	3.30*10 ^*−7*^
*ACTB*	5.51*10 ^*−7*^	9.52*10 ^*-11*^
*MYCN/ACTB*	0.72	3.47*10^3^
**Purification factor** $=\frac{post\;capture\;MYCN/post\;capture\;ACTB}{pre\;capture\;MYCN/pre\;capture\;ACTB}$ = 4.8*10^3^		

A purification factor of 4.8 × 10^3^ was achieved for the *MYCN* RNA as compared to *ACTB* RNA, indicating effective removal of non-target molecules. This finding was confirmed through Nanopore direct-RNA sequencing and transcriptome mapping.

### Nanopore direct RNA sequencing

Sequencing of the RNA library from captured *MYCN* RNA generated 21 POD5 raw sequencing files. After basecalling with Dorado basecaller (v.0.8.2), the uBAM output file contained a total of 20.681 reads, of which 15.127 reads have Phred scores > 7 and read lengths > 100 nucleotides.

**Fig 2:**
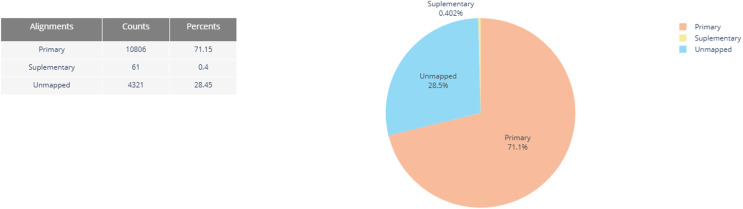
Summary of read alignments mapped to the transcriptome. 71% of basecalled reads with Phred score > 7 and read lengths > 100 were primary mapped to the combined cDNA and ncRNA reference (ENSEMBL v.112). Generated by PycoQC.

Transcriptome mapping, using a combined cDNA and ncRNA reference, resulted in 10.806 primary mapped, 61 supplementary mapped and 4.321 unmapped reads (Fig 2). Quality metrics of basecalled and mapped reads are shown in the step-by-step protocol (S1 File).

Table 2 shows an overview of the most abundant transcripts generated by the sequence alignment software Minimap2 (v.2.26). A total of 7.063 reads mapped to *MYCN* transcripts, corresponding to 65,4% of the primary mapped reads.

**Table 2:** Overview of the transcriptome mapped reads. The table is generated from ‘samtools idxstats’ output. 8 transcripts had more than 100 mapped reads (MYCN-203 transcript also shown).

Transcript ID	Transcript name	Gene	# Reads
ENST00000638417.1	MYCN-202	MYCN	3677
ENST00000281043.4	MYCN-201	MYCN	3350
ENST00000618786.1	RN7SL1–201	RN7SL1	481
ENST00000419083.6	MYCNOS-201	MYCNOS	199
ENST00000641387.2	MYCNOS-203	MYCNOS	177
ENST00000655317.1	novel lncRNA	Lnc-NEMF-1	169
ENST00000659240.1	novel lncRNA	Lnc-NEMF-1	163
ENST00000490232.3	RN7SL2–201	RN7SL2	161
ENST00000703162.1	MYCN-203	MYCN	36

### Detailed investigation of the most abundant primary mapped reads

**Fig 3:**
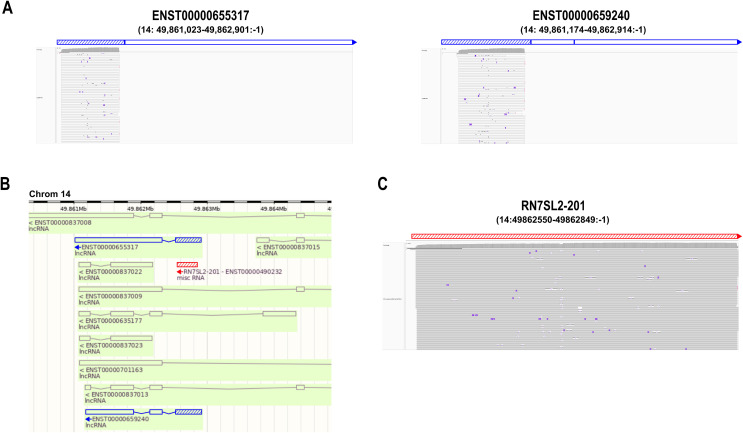
Novel lncRNA reads from the primary transcriptome mapping are RN7SL2-201 transcripts. (A) IGV images of novel lncRNA transcripts ENST00000655317.1 (left) and ENST00000659240.1 (right). The hatched blue area marks the first exon of the corresponding gene ENSG00000282885, also known as lnc-NEMF-1. All mapped reads align to the first exons. (B) Snapshot from ENSEMBL browser of novel lncRNA transcripts (blue, hatched marks first exon). RN7SL2 transcript, which overlap the lncRNA first exons, is marked as red hatched area. (C) IGV alignment of the novel lncRNA reads remapped to the RN7SL2-201 transcript supporting the identity of these reads.

When the 332 reads mapping to the two novel lncRNA transcripts (ENST00000655317.1 and ENST00000659240.1) were viewed in IGV (Fig 3A), we noticed that only part of the first exons (blue hatched) of these transcripts was covered by the reads, and that the exons overlap with the *RN7SL2–201* transcript (Fig 3B, red hatched). In addition, all novel lncRNA reads were multimappers. Extracting the novel lncRNA reads from the uBAM file and remapping them to the *RN7SL2* transcript reveals that all 332 reads are *RN7SL2–201* transcripts (Fig 3C).

We then performed a similar extraction and remapping of all *MYCN* and *MYCNOS* reads and found that most reads map to the main transcripts (*MYCN-201* and *MYCNOS-201*) from these genes. Therefore, after a more detailed investigation of the mapped reads, we concluded that 81.2% of all primary mapped reads derive from 4 genes; *MYCN*, *RN7SL1*, *RN7SL2* and *MYCNOS* (Table 3).

**Table 3:** Final overview of the most abundant primary mapped reads. Two reads mapping uniquely to the MYCN-202 transcript (MYCN, lacking exon 2) are also included (italic).

Transcript ID	Transcript name	Gene	# Reads
ENST00000638417.1	MYCN-201	MYCN	7056
ENST00000490232.3	RN7SL2–201	RN7SL2	492
ENST00000618786.1	RN7SL1–201	RN7SL1	481
ENST00000419083.6	MYCNOS-201	MYCNOS	367
*ENST00000638417.1*	*MYCN-202*	*MYCN*	*2*

### Investigation of unmapped reads

To investigate the identity of the 4321 transcriptome unmapped reads, we first manually inspected the reads in the bam file and noticed many reads consisting of long uninterrupted GAA- and GGT-repeats. These reads constitute 75% of the unmapped reads (3155 and 70 reads with more than five consecutive GAA and GGT, respectively).

The remaining 1096 unmapped reads were aligned to the human genome and rRNA (UCSC: 4V6X_human_rRNAs.fa) references. 363 reads mapped to rRNA genes (28S: 317, 5S: 6 and 18S: 40) and approximately 160 reads mapped to noncoding regions around the *MYCN* gene (5’, introns, or 3’). This leaves us with only 259 reads that we could not identify.

## Discussion

The enrichment protocol is easy to follow and can be used with any target gene. It can be performed in a single workday, and most of the equipment you need is standard laboratory instrumentation and infrastructure.

We have established a gene-specific RNA enrichment protocol employing biotinylated DNA capture probes and streptavidin-coated magnetic beads. Applying this method, we achieved a purification factor of 4,8 × 10^3^ for our target gene, *MYCN*. Nanopore sequencing and subsequent analysis revealed that 65.4% of the primary mapped reads aligned to *MYCN* transcripts, compared to approximately 0.3–0.4% in the absence of enrichment (our unpublished data). A more thorough analysis of all mapped reads showed that 81.2% originated from four genes: *MYCN*, *RN7SL1*, *RN7SL2*, and *MYCNOS*.

Transcriptome mapped reads were evaluated by MAPQ values and visual inspection using IGV. We identified several alignment artifacts. In most cases we observed reads only partially mapping to erroneous transcripts (Fig 3A). This emphasizes the importance of careful evaluation of alignment outputs (compare Tables 2 and 3). However, this is a tedious task and might only be possible with a limited number of transcripts.

One limitation of the method is the large amount of total RNA required to generate sufficient capture RNA. The latest Nanopore direct RNA sequencing kit (SQK-RNA004) recommends 300 ng of poly(A)-tailed RNA or 1 µg of total RNA. However, useful RNA read counts have been achieved using as little as 50 ng input mRNA [2].

In this protocol, the one-gene capture procedure dramatically reduces the amount of RNA available for direct RNA sequencing. We performed a single-step absolute quantification of the capture output RNA using RT-qPCR, employing *in vitro* transcribed full-length *MYCN* RNA to generate the standard curve. Based on this, we estimated the total input for the sequencing library preparation to be approximately 4 pg of RNA (S2 file). Although sequencing can be performed with very small amounts of input RNA, this typically results in decreased output. However, when we compare the reads mapping to the enriched RNA (MYCN) from the enrichment protocol with two non-enriched sequencing runs (RNA from untreated Kelly and CHP-134 cells) we observe more than 10-fold increase in the total number of read counts, confirming improved total enrichment for the gene of interest (S3 File).

The large amount of total RNA required to generate sufficient capture RNA still presents a challenge for applications involving low yield clinical samples, such as patient biopsies. To enable broader use in diagnostic contexts, further refinement of the protocol is therefore necessary. Potential strategies to mitigate low-yield limitations include the use of carrier RNA to minimize sample loss during enrichment, optimizing bead-to-RNA ratios to enhance capture efficiency, and improving RNA recovery methods. Additionally, integrating target RNA amplification prior to sequencing could increase input availability while retaining transcript structure. These adjustments could broaden the applicability of this protocol to clinically relevant low-input samples while maintaining specificity and throughput.

We performed sequencing using the RNA004 sequencing protocol on a MinION flow cell which generated approximately 1.0 GB of output data (~ 20.600 raw reads). For laboratories using the RNA002 sequencing kit (SQK-RNA002), it will be very cost efficient to perform sequencing using the Flongle flow cell (FLO-FLG001) and Flongle sequencing expansion (EXP-FSE001), which can generate up to 2.8 GB data [14,15]. This was, however, not possible with the RNA004 sequencing protocol, since it requires the new RNA004 flow cell (FLO-MIN004RA) not yet available in the Flongle format.

In order to sequence all RNA molecules originating from the *MYCN* oncogene, we added a final polyadenylation step before library preparation. Here, polyA-polymerase artificially extends the tail (3’ end) by adding extra adenosine residues to all RNAs, including polyadenylated mRNAs. While this is a useful step to enhance relevant output, it also presents a significant drawback since information about the native length and natural variations of the poly(A) tail is irretrievably lost. A recent study by Yuan et al. proposes a strategy to address this limitation, using yeast poly(A) polymerase to append short 2′-O-methyladenosine tails that produce distinct nanopore signals and enable comprehensive RNA capture, including organellar transcripts [16].

Extensive experimental handling of RNA samples is well known to cause degradation of the RNA [17]. To evaluate RNA degradation after the enrichment protocol, we analyzed direct RNA sequencing reads, comparing the *MYCN*-capture sample with two non-enriched runs: one from untreated Kelly cells (the same cell line used for capture) and one from the *MYCN*-amplified neuroblastoma cell line CHP-134 (S4 File). The median *MYCN* read length was 392 nt for CapMYCN, compared with 793 nt for Kelly and 805 nt for CHP-134. The reduced median read length clearly indicates that the prolonged RNA handling during the enrichment protocol exposes the RNA to significant gradual degradation. This is also evident when comparing the gene body coverage plots of *MYCN* and *RN7SL1*, and the IGV coverage tracks of *MYCN*, from the same samples (S4 File). To minimize gradual RNA degradation, all handling conditions were carefully controlled by reducing sample exposure time, maintaining low temperatures whenever possible, and minimizing pipetting and vortexing to prevent mechanical stress. Samples were processed promptly and stored under RNase-free conditions to preserve RNA integrity. Although not implemented in this study, RNA degradation could be further minimized by incorporating RNase inhibitors during extraction and handling, and by using DEPC-treated RNase-free water and consumables.

After conducting a more thorough analysis of the mapped reads from sequencing of *MYCN*-enriched RNA, we observed only three transcripts that were significantly expressed (> 100 reads) but not identified as *MYCN* transcripts; *RN7SL1–201*, *RN7SL2–201* and *MYCNOS-201*. *MYCNOS* (*MYCN*-Opposite-Strand) is a long non-coding RNA (lncRNA) transcribed from the *MYCN* locus in the antisense direction overlapping exon1 and part of intron1 of the *MYCN* gene [18]. Krystal et al 1990 demonstrated that most non-polyadenylated RNAs from *MYCNOS* exist in RNA-RNA duplex with *MYCN* mRNAs [19]. They estimated that approximately 5% of *MYCN* mRNAs were duplexed with complementary antisense RNA. These previous observations of *MYCN*-*MYCNOS* RNA duplex formation, most probably explain why we observe *MYCNOS* RNA in *MYCN*-enriched samples.

We have been unable to account for the presence of substantial quantities of RN7SL RNAs in the enriched RNA sample. To the best of our knowledge, no published studies have reported direct interactions between RN7SL RNAs and other RNAs. Also, there appears to be no significant complementarity between the *MYCN* capture probes and *RN7SL1* or *RN7SL2* RNA sequences.

The purity of the RNA sample significantly affects the quality of downstream analyses. Therefore, this enrichment protocol may also serve as a valuable preliminary step for other areas of research, particularly those focusing on a single gene, not limited to RNA sequencing. For instance, long non-coding RNAs (lncRNAs) often exhibit low expression levels and tissue-specific patterns [20]. This approach can therefore enhance the detection of lncRNAs by enriching these transcripts in the tissues or conditions where they are expressed. Additionally, it holds potential for identifying clinically relevant RNA species, such as gene fusion transcripts, which are important diagnostic and prognostic markers in various cancers [21]. Through selective sequencing of disease-specific RNAs in their native form, this protocol offers a powerful tool for transcriptomic profiling in both research and clinical applications.

## Supporting information

S1 File Step-by-step protocol. This protocol is also available on protocols.io. dx.doi.org/10.17504/protocols.io.8epv52m16v1b/v1.
(PDF)

S2 File Absolute quantification of enriched RNA. (PDF)

S3 File Comparison of total number of reads mapping to MYCN from capture protocol and non-enriched control sequencing experiments. (PDF)

S4 File Evaluation of RNA degradation during *MYCN* enrichment protocol. (PDF)

S1 Table Capture probe overview. (XLSX)
