# Supplementary material for: A gene-specific RNA enrichment protocol for nanopore direct-RNA sequencing
Source: PLoS One. 2026 Feb 11;21(2):e0339960. doi: 10.1371/journal.pone.0339960 (PMC12893535; doi:10.1371/journal.pone.0339960)
Supplement: S1 File — This protocol is also available on protocols.io. dx.doi.org/10.17504/protocols.io.8epv52m16v1b/v1. (PDF) [file pone.0339960.s001.pdf]

A gene-specific RNA enrichment protocol for Nanopore Direct RNA sequencing

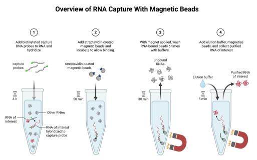

RESERVED DOI:
10.17504/protocols.io.8epv52m16v1b/v1

Maja Bele Dyrendalsli[1], Cecilie Løkke[2], Christer Einvik[1,2]

[1]Department of Pediatrics, Division of Child and Adolescent Health, UNN-University Hospital of North-Norway, Tromsø, Norway.;
[2]Research Group for Child and Adolescents Health, Department of Clinical Medicine, Faculty of Health Science, UiT-The Arctic University of Norway, Tromsø, Norway.

Maja Bele Dyrendalsli
UNN Tromsø: University hospital of North-Norway

Protocol Info: Maja Bele Dyrendalsli, Cecilie Løkke, Christer Einvik . A gene-specific RNA enrichment protocol for Nanopore Direct RNA sequencing . **protocols.io** **https://protocols.io/view/a-gene-specific-rna-enrichment-protocol-for-nanopo-dx6r7rd6**

Created: January 20, 2025

Last Modified: October 13, 2025

Protocol Integer ID: 118705

Keywords: Gene enrichment, RNA sequencing, DNA capture probes, Long-read direct RNA sequencing

Funders Acknowledgements:
The Norwegian Childhood Cancer Society
Grant ID: 220002

Abstract

Oxford Nanopore direct-RNA sequencing, a third-generation sequencing technology, allows for the analysis of native RNA molecules in their natural cellular state. However, cellular RNA is predominantly composed of ribosomal RNA and transcripts from ubiquitously expressed housekeeping genes, which limits the coverage of transcripts from lowly expressed genes. To address this limitation, targeted sequencing can be employed to enrich read coverage by focusing specifically on genes of interest.

Here, we present a step-by-step protocol for gene-specific RNA enrichment followed by Oxford Nanopore direct-RNA sequencing. The enrichment protocol utilizes biotinylated DNA capture probes complementary to the target gene. Following in-solution hybridization of probes to total RNA, a series of stringent washes is applied before elution of the enriched RNA sample. The protocol describes all steps from isolation of cellular total RNA to bioinformatic analyses of raw sequencing data.

As a proof of concept, capture probes were designed to specifically enrich all RNA species encoded by the MYCN oncogene. The enrichment protocol successfully isolated RNAs from the MYCN gene, achieving a purification factor of 4.8×10^3. Direct-RNA sequencing of the enriched RNA sample revealed that 65% of the primary mapped reads aligned to MYCN transcripts. We also include a more thorough analysis of the most abundant non-target mapped reads and unmapped reads.

This protocol proves to be highly effective in removing unwanted RNA species, delivering robust enrichment for the target gene, and significantly enhancing the efficiency of long-read direct-RNA sequencing.

Guidelines

- Work on ice whenever possible. RNA is easily degradable.
- Use RNase-free tubes.
- Prepare a master-mix when working with several reactions. Always make 10% extra, to account for pipetting errors.
- Always use nuclease-free H_2O. Nucleases actively degrade DNA and RNA in samples.
- Avoid excessive vortexing and pipetting, as this can be damaging to the RNA/cDNA molecules.

Materials

General materials

- 0.2 ml PCR tubes
- Thermal cycler
- Heated water bath
- Ice bucket
- Micro centrifuge
- Hula mixer
- Magnetic rack for 0.2 ml tubes
- Agencourt SPRIstand magnetic tube
- 1.7 ml RNase-free tubes
- Nuclease-free water
- Timer
- 70% ethanol, freshly prepared with nuclease-free H_2O.

For quality control

- Agilent 2100 bioanalyzer
- Agilent RNA 6000 Nano reagents kit
- RNaseZAP

For the capture

- Oligonucleotide capture probes specific to your target gene
- Total RNA
- Agencourt RNA clean XP beads (Beckman Coulter)
- IDT XGen^{TM} Hybridization and wash kit (catalog no. 1080577)\
- Dynabeads
- 1.7 mL low-bind tubes

For the RNA sequencing

Nanopore has provided a list of the necessary equipment and consumables.
Nanopore dRNA sequencing equipment and consumables

Troubleshooting

Safety warnings

Please refer to the safety warnings provided by the manufacturers of the individual reagents and chemicals.

Before start

Make sure you are working in an RNase-free environment. Clean your workspace, make sure you have all the necessary equipment and prepare an ice bucket.

Overview of Capture protocol

1 Overview of the procedures in the Capture protocol.

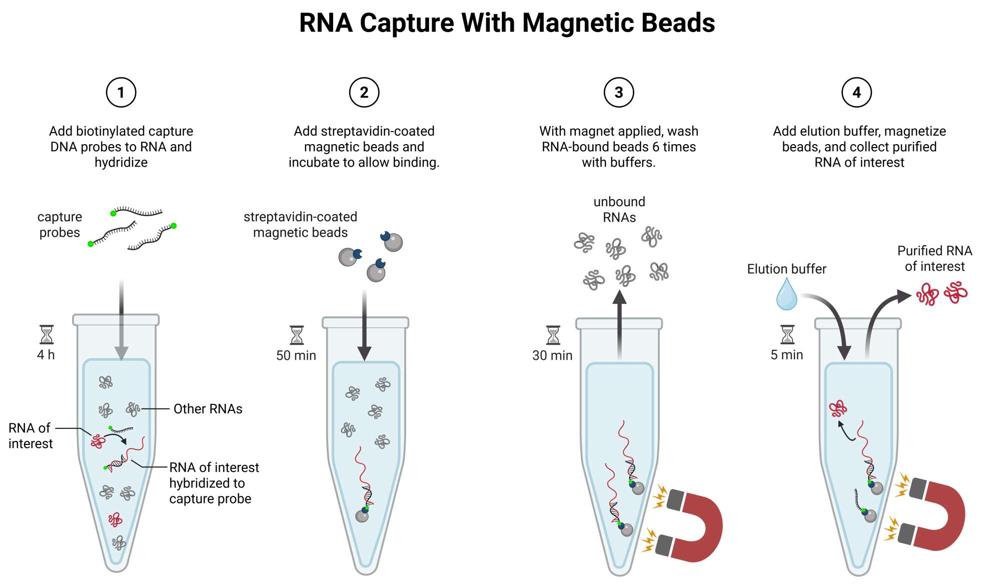

Figure 1: Overview of the procedures in the Capture protocol.

Capture probe design

2 Design and order biotinylated DNA capture probes complementary to the target RNA.

60-nt uniform 2X tiling biotinylated capture probes were ordered from IDT, Integrated DNA Technologies. The probes were designed as antisense DNA oligonucleotides covering the complete MYCN gene sequence twice (chr2:15.940.550-15.946.097, genome build: Hg38). 12 probes covering intron 2 did not pass the IDT proprietary quality control system and were excluded from the mixture (Figure 2). The 178 probes were pooled and dissolved in nuclease-free water to final concentration: 200amol/probe/ul.

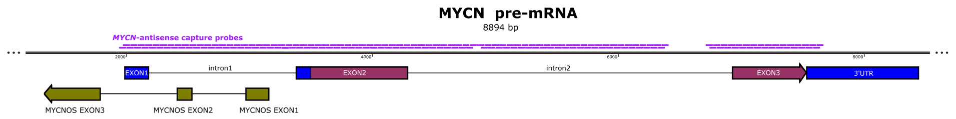

Figure 2: Overview of location and coverage of the 178 capture probes on schematic presentation of the MYCN gene. MYCNOS (MYCN Opposite Strand) is also shown. Figure is drawn to scale.

Isolation of total RNA and quality control

3 Extract total RNA from cells.

We do this according to the protocol adapted by Untergasser, which integrates TRIzol-based lysis with RNeasy Mini spin column purification. Link: **Untergasser RNA isolation protocol**

3.1 Measure the RNA concentration in the sample.

We obtained a concentration of approximately 1220 ng/µL with a total collected volume of 175 µL, derived from each of five separate T75 cell culture flasks. The concentration was measured using Nanodrop. Fluorometric assays, such as Qubit RNA, can also be used for the quantification of RNA. It is more accurate than Nanodrop for low concentrations and in the presence of contaminants.

3.2 Check the quality of the RNA. RNA integrity was assessed on an Agilent 2100 Bioanalyzer. Samples consistently showed RIN values between 9 and 10, indicating intact RNA suitable for RNA-seq. A RIN ≥ 7 is generally considered sufficient for transcriptomic applications (Fleige & Pfaffl, 2006; Schroeder et al., 2006).

Prepare for hybridization

4 Prepare the hybridization buffer according to Table 1.

Component	Volume [µL]
XGen 2X Hyb. Buffer	17
XGen 2X Hyb. Buffer enhancer	5.4
MYCN-antisense capture probes	4.0
Nuclease-free H2O	7.6
**Total**	**34**

Table 1: Hybridization buffer components

5 Perform a cleanup. Elute the RNA sample in 34 µL hybridization buffer.

Cleanup was performed using Agencourt RNAClean XP (single tube format), a solid-phase reversible immobilization (SPRI)–based magnetic bead system optimized for RNA. We added 1.8 volumes of RNAClean XP relative to the reaction volume (e.g., 180 µL for a 100 µL reaction).

Hybridization

6 Transfer the sample to 0.2 ml PCR tubes.

7 Perform the hybridization program (Table 2) in a thermal cycler. The temperature of the lid should be 100 °C . The default ramp rate was used.

Temperature [C]	Time
95	30 sec
65	4 hours
65	hold

Table 2: Hybridization program

8 With 1 hour left of the hybridization program, let the Dynabeads equilibrate to room temperature.

Bead wash

1m 30s

9 Prepare dilutions of wash buffers and bead resuspension mix according to Tables 3 and 4, respectively. Buffers are provided in the IDT xGen hybridization and wash kit (catalog number: 1080577).

Buffer	Buffer volume [µL]	H2O volume [µL]
Wash buffer 1	84	756
Wash buffer 2	48	432
Wash buffer 3	48	432
Bead wash buffer	480	480
Stringent wash buffer	96	864

Table 3: Wash buffers

Component	Volume per reaction [µL]
xGen 2x hybridization Buffer	8.5
xGen 2x hybridization Buffer Enhancer	2.7
Nuclease-free water	5.8
**Total**	**17**

Table 4: Bead resuspension mix

10 330 µL of wash buffer 1 is transferred into a separate tube, so that it can later be placed in a heated water bath.

11 Distribute stringent wash buffer into two tubes of 480 µL each.

12 Mix the dynabeads thoroughly by vortexing for 00:00:15 . 15s

12.1 Transfer 50 µL of dynabeads per sample into a single 1.7 mL low-bind tube. For example, for 1 capture, prepare 50 µL of beads and for 2 captures, prepare 100 µL of beads.

12.2 Perform the following wash: 1m

a. Add 100 µL of bead wash buffer per capture, then pipette mix 10 times.

b. Place the tube on a magnetic rack for approximately 00:01:00 , allowing the beads to fully separate from the supernatant.

c. Carefully remove and discard the supernatant.

12.3 Repeat step 12.2 twice, for a total of 3 washes.

12.4 Resuspend the beads in 17 µL of bead resuspension mix per sample.

12.5 Mix thoroughly to ensure that the beads are not left to dry in the tube. If needed, briefly centrifuge the tube at 25 x g.

Bead capture

1h

13 Place the stringent wash buffer tubes, as well as one of the wash buffer 1 tubes, in a water bath of 65 °C , along with 100 µL H_2O in a tube.

13.1 After the 4-hour incubation, remove the tubes with hybridization mix (step 7) from the thermal cycler. Transfer 17 µL of resuspended streptavidin beads to the tube with hybridization mix (RNA + probe). Resuspend the beads in the hybridization mix.

13.2 Vortex to ensure that the sample is fully resuspended. Gently and briefly centrifuge, if needed (10 sec at 25 x g).

13.3 Place the sample tube in a thermal cycler set to 65 °C (lid: 70 °C) and incubate for 45 minutes (00:45:00).
Alternatively, a thermal shaker can be used. If a thermal shaker is used, proceed directly to Step 13.5 and omit Step 13.4. 45m

13.4 Every 10-12 min, remove the tube from the thermal cycler and gently vortex to ensure that the sample is fully resuspended.

13.5 At the end of the 45 min, take the sample off the thermal cycler. Proceed immediately to *Heated washes*.

Heated washes

13m

14 Transfer 100 µL of heated Wash buffer 1 (from the tube in the water bath) to the sample. Pipette mix 10 times.

> **Note**
>
> Try to minimize bubble formation.

14.1 Place the tube on a magnetic rack for 00:01:00 . Remove the supernatant. 1m

14.2 Remove the tube from the magnet and add 150 µL of heated stringent wash buffer to the sample.

14.3 Pipette mix 10 times.

> **Note**
>
> Be careful not to introduce any bubbles.

14.4 Incubate the sample in the water bath at 65 °C for 00:05:00 . 5m

14.5 Place the sample on the magnet for 00:01:00 . Carefully remove the supernatant. 1m

14.6 Remove the tube from the magnet and add 150 µL of heated stringent wash buffer to the sample.

14.7 Pipette mix 10 times.

> **Note**
>
> Be careful not to introduce any bubbles.

14.8 Incubate the sample in the water bath at 65 °C for 00:05:00 . 5m

14.9 Place the tube on a magnet for 00:01:00 . 1m

Room temperature washes 9m 30s

15 Remove and discard the supernatant. Add 150 µL of wash buffer 1 equilibrated to room temperature.

15.1 Vortex thoroughly until fully resuspended.

15.2 Incubate for 00:02:00 while alternating between vortexing for 30 sec and resting for 30 sec, to ensure the mixture remains homogenous. 2m 30s

15.3 At the end of the incubation, briefly centrifuge the tube.

15.4 Place the sample on the magnet for 00:01:00 . 1m

15.5 Remove the supernatant. Add 150 µL wash buffer 2.

15.6 Vortex thoroughly until fully resuspended.

15.7 Incubate for 00:02:00 while alternating between vortexing for 30 sec and resting for 30 sec, to ensure the mixture remains homogenous. 2m

15.8 At the end of the incubation, briefly centrifuge the tube.

15.9 Place on the magnet for 00:01:00 . 1m

15.10 Remove the supernatant. Add 150 µL of wash buffer 3.

15.11 Vortex thoroughly until fully resuspended.

15.12 Incubate for 00:02:00 while alternating between vortexing for 30 sec and resting for 30 sec, to ensure the mixture remains homogenous. 2m

15.13 At the end of the incubation, briefly centrifuge the tube.

15.14 Place the sample tube on the magnet for 00:01:00 . 1m

15.15 Carefully remove and discard the supernatant.

15.16 With the sample tube still on the magnet, use a fresh pipette tip to remove residual wash buffer 3 from the tube, then remove the tube from the magnet.

Sample elution

2m

16 Elute each sample in 20 µL heated H_2O (from the tube in the water bath).

17 Pipette mix 10 times to resuspend any beads stuck to the side of the tube.

18 Incubate the sample for 00:02:00 after the addition of water.

2m

19 Resuspend the sample and place on a magnet.

20 Collect the purified sample using a pipette. Freeze at -80 °C or proceed to the next step immediately.

Optional: (to include non-polyA RNAs)

30m

21 Oxford Nanopore direct RNA (dRNA) sequencing kits require a 3′ polyadenylated (poly(A)) tail on template molecules for successful sequencing library preparation. To include non-polyadenylated RNAs from the enriched sample, an in vitro polyadenylation step can be added. Due to low concentration of the sample, the most practical cleanups are magnetic-bead-based cleanup kits, or column-based cleanup kits with low-elution-volumes.

30m

We used the following kit by New England Biolabs: NEB #M0276. Components needed are listed in Table 5. This step should be followed by a clean-up procedure.

Component	Volume
RNA	15 µl enriched RNA (approx. 4 pg)
10X E. coli Poly(A) Polymerase Reaction Buffer	2 µl (1X)
ATP	2 µl
E.coli Poly(A) Polymerase	1 µl

Component	Volume
Total	20 µl

Table 5: Components needed for Poly(A) Tailing of RNA using NEB #M0276 kit.

1. Add components (in the specified order)
2. Incubate for 00:30:00 at 37 °C *
3. Perform a cleanup. Cleanup was performed using Agencourt RNAClean XP (single tube format), a solid-phase reversible immobilization (SPRI)–based magnetic bead system optimized for RNA. We added 1.8 volumes of RNAClean XP relative to the reaction volume (e.g., 180 µL for a 100 µL reaction).
Due to low concentration of the sample, the most practical cleanups are magnetic-bead-based cleanup kits, or column-based cleanup kits with low-elution-volumes.

* A shorter incubation time might be beneficial, as documented by Oxford Nanopore technologies. They recommend an incubation time of 0.5-1.5 minutes, with a maximum E. coli poly(A) polymerase reaction incubation time of 5 minutes. They also report that incubation times longer than 5 minutes may result in lower total sequencing yields.
https://nanoporetech.com/document/requirements/polyad-npolyA-ecoli

Validation of gene enrichment

22 To validate and quantify the target gene enrichment, RT-qPCR should be performed on the sample before and after the enrichment is carried out. Use Beta-actin (ACTB) or another gene as a control gene for comparison. The purification factor of the target gene can then be calculated using the N0 values and the ratio between the target gene and the control gene, before and after the enrichment.

We synthesized cDNA using a random hexamer primer and Multiscribe Reverse Transcriptase (Invitrogen), according to the manufacturer's protocol. Each qPCR reaction (20 µL) contained 5 µL cDNA (10 ng pre-capture or 3 µL post capture + 2 µL nucleasefree H_2O), 10 µL of SYBR Green PCR Master Mix (Thermo Fisher) and 0.8 µL of each forward and reverse primers.

Primers used for the qPCR are listed below in Table 6.

Oligo	Primer sequence
ACTB forward	5'-TCACCCACACTGTGCCCATCTACGA-3'
ACTB reverse	5'-CAGCGGAACCGCTCATTGCCAATGG-3'
MYCN forward	5'-AAGAACCCAGACCTCGAGTT-3'
MYCN reverse	5'-CAGCAGCTCAAACTTCTTCCA-3'

Table 6: Primers used for qPCR.

Library preparation

23 Prepare the library according to the Nanopore Direct RNA sequencing protocol (SQK-RNA004). Link: **Nanopore direct RNA sequencing (SQK-RNA004) protocol**.

Note

The RNA CS is only necessary if you will be comparing two sequencing runs. Otherwise it can be omitted.

Note

Remember to take the flow cells out of the fridge 30-60 min before use.

Nanopore dRNA sequencing

24 Prime and load the flow cell according to the Nanopore Direct RNA sequencing protocol (SQK-RNA004). Link: **Nanopore direct RNA sequencing (SQK-RNA004) protocol**

25 Perform sequencing using a GridIon Mk1 (MinKNOW 24.02.16).

Suggestions for bioinformatics analysis of the raw sequencing data

26 Basecalling and mapping were performed on resources provided by Sigma2 - the National Infrastructure for High-Performance Computing and Data Storage in Norway. NVIDIA A100 GPU graphics card was used for basecalling.

Nanopore dRNA sequencing outputs raw sequencing data in **POD5 format**.

27 **Basecalling:**
Software: **Dorado basecaller** (v. 0.8.2+6b413c9) with the following settings:
Model: rna004_130bps_sup@v5.0.0
Input: directory contaning raw POD5 sequencing files.

```
dorado basecaller --verbose --min-qscore 7 \
/Dorado_models_folder/rna004_130bps_sup@v5.1.0 \
pod5_folder/ > capMYCN.ubam
```

Other settings:
If basecalling with all available modified bases:
--modified-bases inosine_m6A m5C pseU
If basecalling to output polyA tail lengths (pt:i tag)
--estimate-poly-a

Dorado basecaller outputs unmapped BAM files (uBAM).

- remove reads shorter than 100 nt.

```
samtools view -e 'length(seq)>100'  -O BAM -o output.uBAM
input.BAM
```

28 **Convert uBAM to fastq for mapping:**
Software: Samtools (v. 1.18)

```
samtools fastq -T '*' (input.uBAM) > (output.fastq)
```

29 **Mapping:**
Software: Minimap2 (v.2.26)

- mapping to genome: (human hg38 - ENSEMBL v.112)

```
minimap2 -ax splice -uf -k14 -t16 -y --secondary=no
(reference_genome.fasta) (fastq file) > (output_DNA.sam)
```

- mapping to transcriptome: (human hg38 - ENSEMBL v.112, combined cDNA and ncRNA reference)

```
minimap2 -ax map-ont -t16 -y --secondary=no
(reference_transcriptome.fasta) (fastq file) > (output_cDNA.sam)
```

30 **Generate coordinate sorted BAM files with cooresponding indexes:**
Minimap2 output alignments in **SAM format**, which is converted to **BAM format**, coordinate sorted and indexed using Samtools:

- convert to BAM:

```
samtools view -@16 -Sb -o (output.bam) (input.sam)
```

- sorting BAM file:

```
samtools sort -@16 -O BAM -o(output_sorted.bam) (input.bam)
```

- indexing sorted BAM file:

```
samtools index -@16 (sorted BAM file)
```

31 **Other bioinformatics:** Quality Control
ToulligQC(v2.7.1) and **pycoQC**(v2.5.2) were used to assess the quality of raw and mapped reads, respectively.

- ToulligQC was used to analyze raw reads.

```
toulligqc --thread 7 -a sequencing_summary_file.txt  --qscore-threshold 7 --report-name (report name) -o toulligQC_report_.html
```

- pycoQC was used to analyze raw and mapped reads.

```
pycoQC -f sequencing_summary_file.txt -a (genome-mapped bam file) --report_title (report name) -o pycoQC_report.html
```

Results and interpretation

32 **Capture yield quantification**
RT-qPCR was used to quantify the purification of *MYCN* RNAs in the sample, with beta-actin (*ACTB*) as a control gene.The purification factor was calculated using the

relationship between *MYCN* and *ACTB* pre- and post-capture. These values are listed in Table 7. The purification factor was $4.8*10^3$, making the enrichment highly successful.

	Pre capture	Post capture
MYCN	$3.96*10^{-7}$	$3.30*10^{-7}$
ACTB	$5.51*10^{-7}$	$9.52*10^{-11}$
MYCN/ACTB	0.72	$3.47*10^{3}$

$$\textbf{Purification factor} = \frac{post\ capture\ MYCN/post\ capture\ ACTB}{pre\ capture\ MYCN/pre\ capture\ ACTB} = \mathbf{4.8*10^3}$$

Table 7: Calculations of the purification factor. The second and third columns show N0 values (italic) and relations. N0 is the qPCR efficiency-corrected target quantity as reported by LinRegPCR (v.2021.2). The final purification factor is shown in bold.

33 **Sequencing Quality Control**

Below are some metrics and plots generated by ToulligQC and pycoQC.

Status	# Reads	Median Read Length	Median PHRED score
All reads	20681	306	14.45
Pass reads	16032	334	16.10
Fail reads	4649	164	3.33

Table 8: Basecall summary from ToulligQC and pycoQC. Pass reads have PHRED score >7.

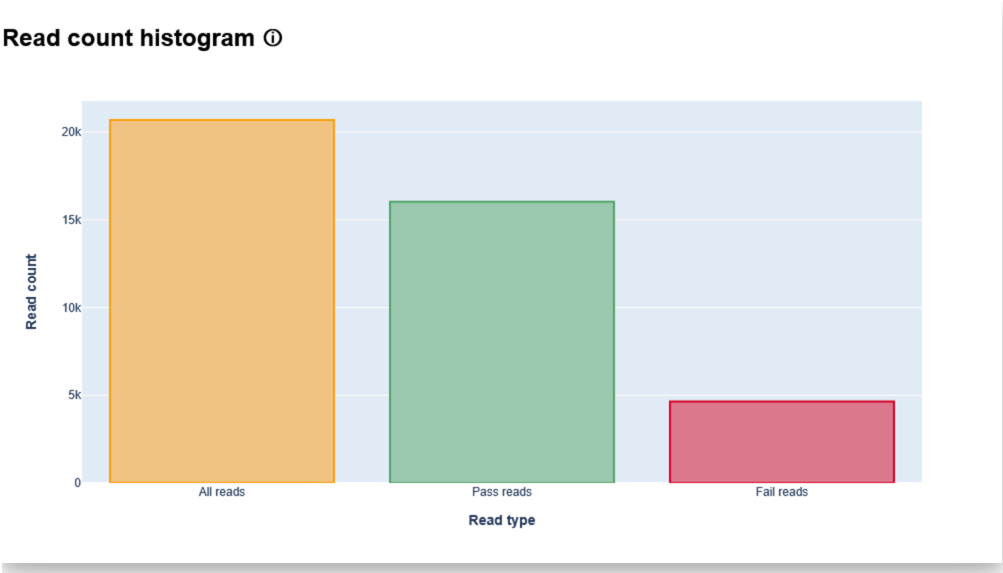

Figure 2: Read count histogram generated by ToulligQC. Pass reads have PHRED score > 7.

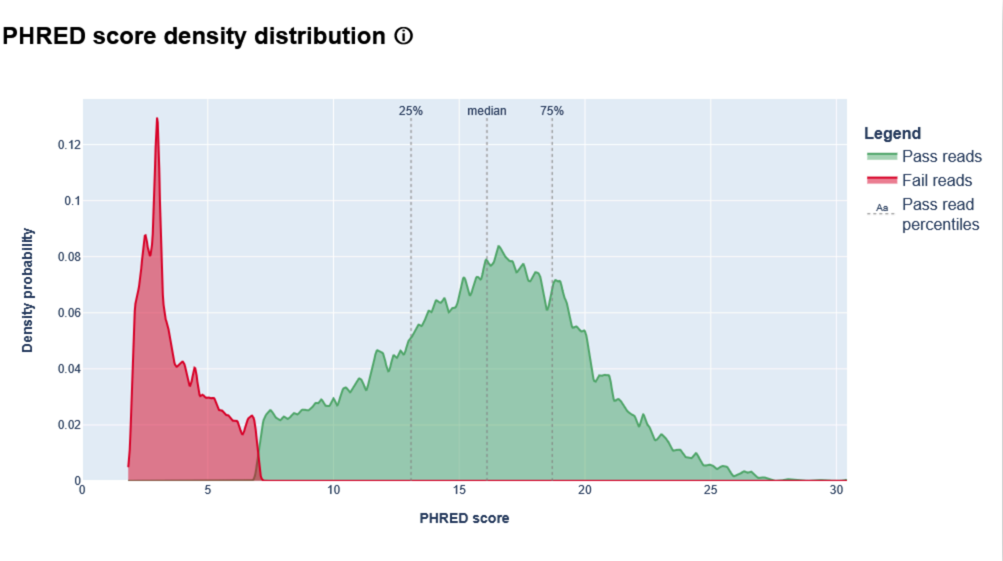

Figure 3: Distribution of mean base q-scores generated by ToulligQC. Pass reads have PHRED scores >7.

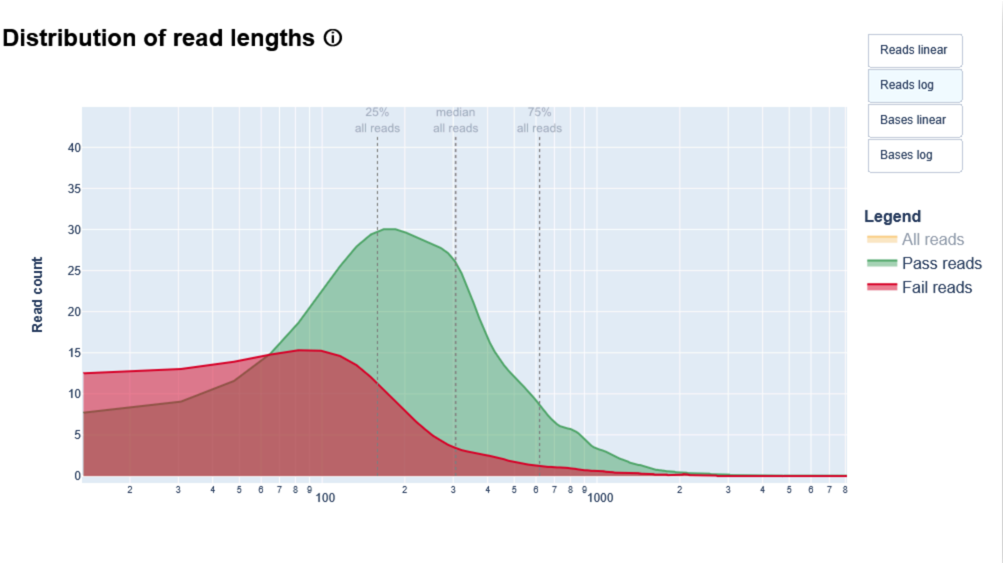

Figure 4: Distribution of basecalled read lengths generated by ToulligQC. Pass reads have PHRED score > 7. Three vertical lines show median, first and third quartiles of all reads (pass + fail).

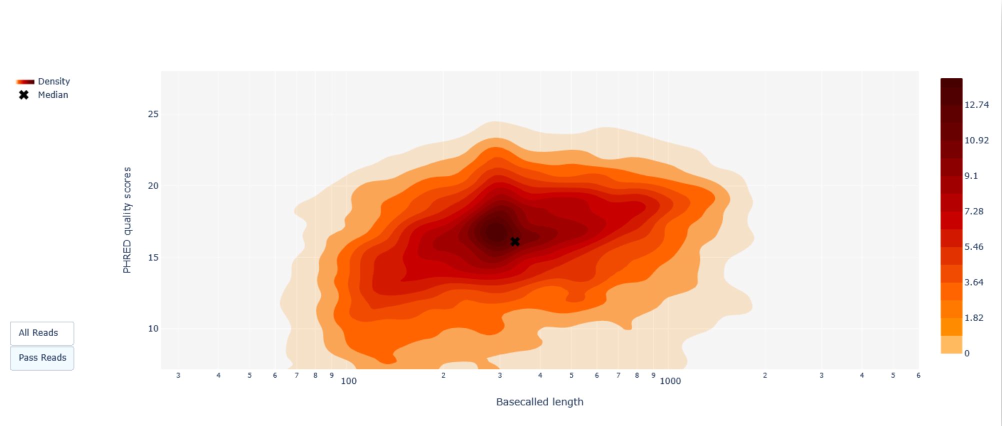

Figure 5: Basecalled reads length vs reads PHRED quality generated by pycoQC. Pass reads (PHRED score >7) are shown.

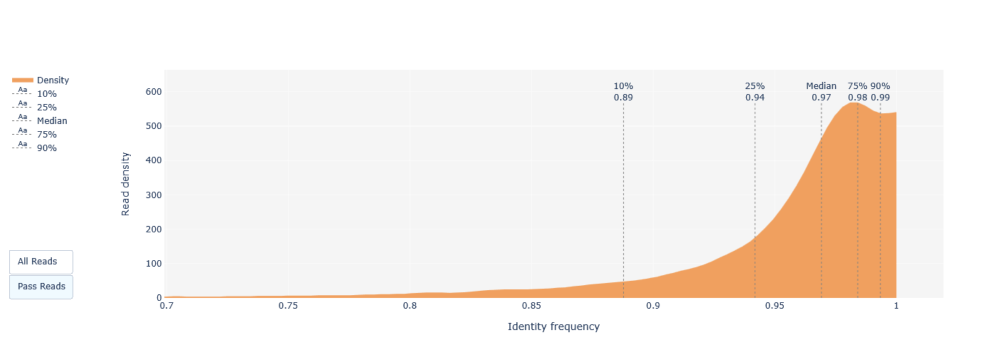

Figure 6: Aligned read identity generated by pycoQC.

34 **Summary of sequencing data:**

Sequencing generated 21 POD5 files. Basecalling generated a total of 20.681 raw sequencing reads in the uBAM file, of which 15.127 reads have phred scores >7 and read lengths > 100 nt.

Mapping ('phred score' >7 and 'read lengths' > 100 nt)
Transcriptome (combined cDNA and ncRNA reference) mapping resulted in 10.806 primary mapped, 61 supplementary mapped and 4321 unmapped reads. 7.063 reads mapped to MYCN transcripts (Table 9).

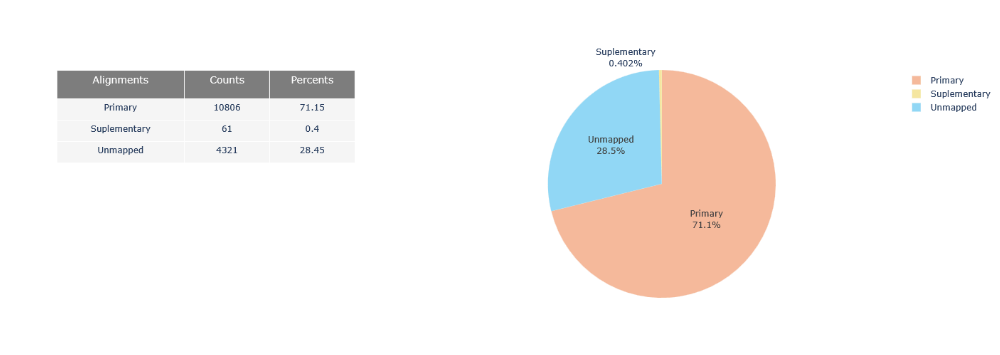

Alignments	Counts	Percents
Primary	10806	71.15
Suplementary	61	0.4
Unmapped	4321	28.45

Figure 7: Summary of reads alignment generated by pycoQC.

Table 9 shows an overview of transcripts with more than 100 mapped reads, along with all three MYCN transcripts. The eight transcripts are encoded by 5 genes and account for 77.5 % of all mapped reads.

Transcript ID	Transcript name	Gene	# reads
ENST00000638417.1	MYCN-202	MYCN	3677
ENST00000281043.4	MYCN-201	MYCN	3350
ENST00000618786.1	RN7SL1-201	RN7SL1	481
ENST00000419083.6	MYCNOS-201	MYCNOS	199
ENST00000641387.2	MYCNOS-203	MYCNOS	177
ENST00000655317.1	novel lncRNA	Lnc-NEMF-1	169
ENST00000659240.1	novel lncRNA	Lnc-NEMF-1	163
ENST00000490232.3	RN7SL2-201	RN7SL2	161
ENST00000703162.1	MYCN-203	MYCN	36

Table 9: Eight transcripts had more than 100 mapped reads (MYCN-203 transcript also shown).

A more thorough analysis of the most highly expressed mapped reads can be viewed in the peer-reviewed article interlinked with this protocol.

Limitations and challenges

35 A limitation to the method is the great amount of total RNA needed to produce the necessary amount of capture RNA. The latest version of Nanopore direct RNA sequencing, SQK-RNA004, calls for 300 ng of poly(A) tailed RNA or 1 µg of total RNA. The sequencing can still be performed with lower amounts of sample. However, there will be a decrease in output.

A key challenge in RNA-based experiments is the inherent susceptibility of RNA to degradation by RNases. Although we did not include RNase inhibitors in our experiments, their use could be beneficial for preserving RNA integrity, particularly when working with sensitive or low-abundance samples. It should be noted, however, that many RNase inhibitors are thermolabile, and their protective effect may be reduced at the elevated temperatures used during hybridization. In addition, minimizing the time between RNA isolation and downstream processing can help reduce degradation.

Acknowledgements

The authors are grateful to senior engineer Hagar Taman at the Genomic Support Center in Tromsø (GSCT) and senior researcher Christopher Graham Fenton at the Computational Biology Research Group (CBRG), Department of Clinical Medicine at UiT-The Arctic University of Norway, for their assistance during Oxford Nanopore Direct-RNA sequencing and bioinformatic analyses. The authors further thank IDT- Integrated DNA Technologies for their help during probe design and establishment of this protocol.
