# Supplementary material for: A gene-specific RNA enrichment protocol for nanopore direct-RNA sequencing
Source: PLoS One. 2026 Feb 11;21(2):e0339960. doi: 10.1371/journal.pone.0339960 (PMC12893535; doi:10.1371/journal.pone.0339960)
Supplement: S2 File — (PDF) [file pone.0339960.s002.pdf]

S2 File. Absolute quantification of enriched RNA.

Absolute quantification of *MYCN* RNA was carried out using the SuperScript™ III One-Step RT-PCR System with Platinum™ Taq DNA Polymerase (Invitrogen). Here, reverse transcription and PCR are performed in a single step for efficient and sensitive absolute quantification of RNA.

Preparation of *in vitro* transcribed full-length *MYCN* RNA:

XbaI-linearized *MYCN*_TrueClone (sc116780 – Origene) plasmid was used as template for the *in vitro* run-off transcription reaction. The plasmid is an untagged clone of *MYCN* cDNA cloned into the T7 RNA polymerase-containing vector pCMV6-XL4. XbaI is a unique restriction enzyme located in the polylinker downstream of *MYCN* cDNA. We used MEGAscript™ T7 Transcription Kit (Invitrogen) to make a 2496 nt *MYCN* RNA (MYCN_IVT_RNA) corresponding to full-length *MYCN* mRNA. TURBO DNase (Invitrogen) was added to the reaction to remove the template DNA. RNA concentration was determined using the Qubit Fluorometer (RNA HS Assay Kit - Invitrogen), and RNA integrity was assessed with the Agilent 2100 Bioanalyzer (RNA 6000 nano assay – Agilent).

Both MYCN_IVT_RNA (serial dilutions) and the enriched MYCN RNA (Capture_RNA) was amplified using the SuperScript™ III One-Step RT-PCR System with Platinum™ Taq DNA Polymerase kit with MYCN-specific primers MYCN_forward (5'-AAGAACCCAGACCTCGAGTT-3') and MYCN_reverse (5'-CAGCAGCTCAAACTTCTTCCA-3'), as recommended by the manufacturer.

A standard curve was generated from a serial dilution of MYCN_IVT_RNA of known concentrations (table below).

Sample name	Cq value	RNA input (pg)*	Log RNA input
MYCN_IVT_RNA	6.57	128	2.11
MYCN_IVT_RNA	10.07	12.8	1.11
MYCN_IVT_RNA	13.94	1.28	0.10720997
MYCN_IVT_RNA	17.74	0.128	-0.89279003
MYCN_IVT_RNA	20.99	0.0128	-1.89279003
MYCN_IVT_RNA	22.94	0.00128	-2.89279003
Capture_RNA	15.23	0.446684	-0.349975

* Quantity of template (MYCN IVT RNA) input for one-step RT-qPCR reaction.

The Cq values from MYCN_IVT_RNA were plotted against the known concentrations to establish the standard curve (figure below), which was subsequently used to determine the *MYCN* RNA concentration in the enriched sample.

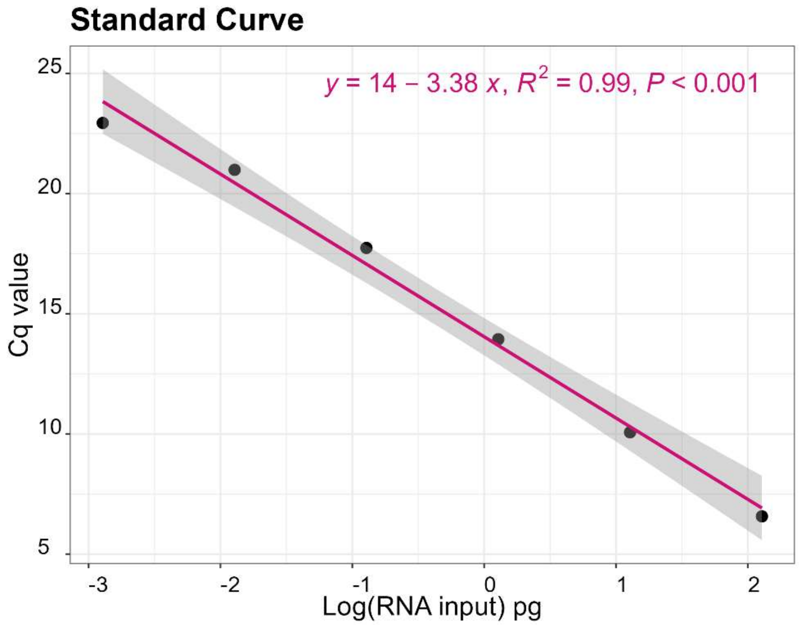

Data from the table above was used for plotting with R (v.4.4.1) packages 'ggplot2' (v.3.5.1) and 'ggmisc' (v.0.6.1). A linear model (lm) function was used for line fitting with level of confidence interval set to 0.95.

A total of 8.5 µL of capture RNA was used for library preparation. At a concentration of 0.45 pg/µL, this corresponds to approximately 4 pg of input RNA.
