# Supplementary material for: A gene-specific RNA enrichment protocol for nanopore direct-RNA sequencing
Source: PLoS One. 2026 Feb 11;21(2):e0339960. doi: 10.1371/journal.pone.0339960 (PMC12893535; doi:10.1371/journal.pone.0339960)
Supplement: S3 File — (PDF) [file pone.0339960.s003.pdf]

S3 File. Comparison of total number of reads mapping to *MYCN* from capture protocol and non-enriched control sequencing experiments

We compared the total number of reads mapping to *MYCN* transcripts (MYCN-201, MYCN-202 and MYCN-203) from the capture protocol ('CapMYCN_Kelly') with two different non-enriched control direct-RNA004 sequencing experiments. One sample is from untreated Kelly cells (same cell line as used for the capture protocol – 'noCap_Kelly'), while the other is from the *MYCN*-amplified neuroblastoma cell line CHP-134 ('noCap_CHP'). Kelly cells have been reported to contain approx. 1,6x higher *MYCN* expression compared to CHP-134 (De Preter et al 2004). As shown in the table below, the enrichment protocol significantly enhanced (> 10x) the total number of reads mapping the gene of interest (total *MYCN* transcripts).

Sample:	Total transcriptome mapped $	Primary mapped (-F2308)	MYCN-201	MYCN-202	MYCN-203	Total MYCN transcript	MYCN % total	MYCN % of primary mapped
CapMYCN_Kelly	15.127	10.806	3.350	3.677	36	**7063**	46,7 %	65,4 %
noCap_Kelly	1.731.197	950.519	373	326	3	**702**	0,04 %	0,07 %
noCap_CHP	1.363.628	980.161	203	163	5	**371**	0,03 %	0,04 %

($ qscore>7, read_length>100)

Samtools view and *samtools idxstats* were used to extract number of reads from transcriptome mapped bam files.

# Subset files to include only reads >100 nt:

samtools view -e 'length(seq)>100' -O BAM -o (transcriptome_mapped)_over100.bam (transcriptome_mapped)_sort.bam

# Extract number of primary mapped reads:

samtools view -c -F2308 (transcriptome_mapped)_over100.bam

# Extract number of MYCN transcripts

samtools idxstats (transcriptome_mapped)_over100.bam | grep 'ENST00000281043.4' ***(# MYCN-201)***

samtools idxstats (transcriptome_mapped)_over100.bam | grep 'ENST00000638417.1' ***(# MYCN-202)***

samtools idxstats (transcriptome_mapped)_over100.bam | grep 'ENST00000703162.1' ***(# MYCN-203)***

Reference:

De Preter K, Pattyn F, Berx G, et al. Combined subtractive cDNA cloning and array CGH: an efficient approach for identification of overexpressed genes in DNA amplicons. BMC Genomics. 2004 Feb;5(1):11. DOI: 10.1186/1471-2164-5-11. PMID: 15018647; PMCID: PMC365025.
