# Supplementary material for: A gene-specific RNA enrichment protocol for nanopore direct-RNA sequencing
Source: PLoS One. 2026 Feb 11;21(2):e0339960. doi: 10.1371/journal.pone.0339960 (PMC12893535; doi:10.1371/journal.pone.0339960)
Supplement: S4 File — (PDF) [file pone.0339960.s004.pdf]

S4 File. Evaluation of RNA degradation during *MYCN* enrichment protocol.

Extensive handling of RNA is well known to cause gradual degradation (Wang et al 2012). Therefore, we evaluated RNA degradation after the capture protocol by analyzing the direct RNA sequencing reads. We compared the reads from *MYCN* capture with two non-enriched sequencing runs. One sample is from untreated Kelly cells (same cell line as used for the Capture protocol), while the other is from the *MYCN*-amplified neuroblastoma cell line CHP-134.

We calculated the median and mean read lengths for MYCN-201 and MYCN-202 annotated transcripts in the enriched sample (CapMYCN) as well as the two non-enriched samples (Kelly and CHP-134).

The median and mean read lengths were calculated as follow;

```
# Extract Reads Mapping to Both MYCN Transcripts (not MYCN-203)
samtools view (transcriptome_mapped)_sort.bam ENST00000281043.4 ENST00000638417.1 > filtered_reads.sam

# Extract Read Lengths
awk '{if($1 !~ /^@/) print length($10)}' filtered_reads.sam > read_lengths.txt

# Compute the Median
sort -n read_lengths.txt | awk '{a[NR]=$1} END{if(NR%2){print "Median Read Length: " a[(NR+1)/2]} else {print "Median Read Length: "
(a[NR/2]+a[NR/2+1])/2}}'

# Compute the Mean
awk '{sum+=$1} END {print "Mean Read Length: " sum/NR}' read_lengths.txt
```

Sample	Median *MYCN* read length	Mean *MYCN* read length
CapMYCN	392	505,6
Kelly	793	952,6
CHP-134	805	966,6

The reduced median read lengths for enriched *MYCN* transcripts clearly indicate that the prolonged RNA handling during the capture protocol exposes the RNA to gradual degradation.

This is further illustrated by gene coverage plots of *MYCN* gene (chr2:15938396-15947022) and *RN7SL1* gene (chr14:49586580-49586878) and the IGV coverage tracks from these samples, shown below. *RN7SL1* was included because it is among the transcripts most highly co-enriched with *MYCN* transcripts during the capture protocol.

The GeneBody coverage plot was made as follow;

1) **Genomic regions corresponding to MYCN gene (Chr2:15938396-15947022) and RN7SL1 gene (Chr14:49586580-49586878) were extracted from the genome mapped sorted bam files using samtools.**

samtools view -b -h (genome_mapped)_sort.bam 2:15938396-15947022 > (genome_mapped)_MYCN.bam
samtools view -b -h (genome_mapped)_sort.bam 14:49586580-49586878 > (genome_mapped)_RN7SL1.bam

2) **The MYCN and RN7SL1 bam files were indexed using samtools index**

samtools index input.bam

3) **Gene Body Coverage was calculated using 'geneBody_coverage.py' version 5.0.2 from the RSeQC package (Wang et al. 2012).**

geneBody_coverage.py -i (indexed_bam_files) -r (Reference_bed_file) -l 100 -f pdf -o MYCN_RN7SL1_geneBody_coverage_output

4) **The 'geneBody.R' output file, containing the coverage data, was imported to R for custom plotting using 'ggplot2'.**

GeneBody Coverage plot:

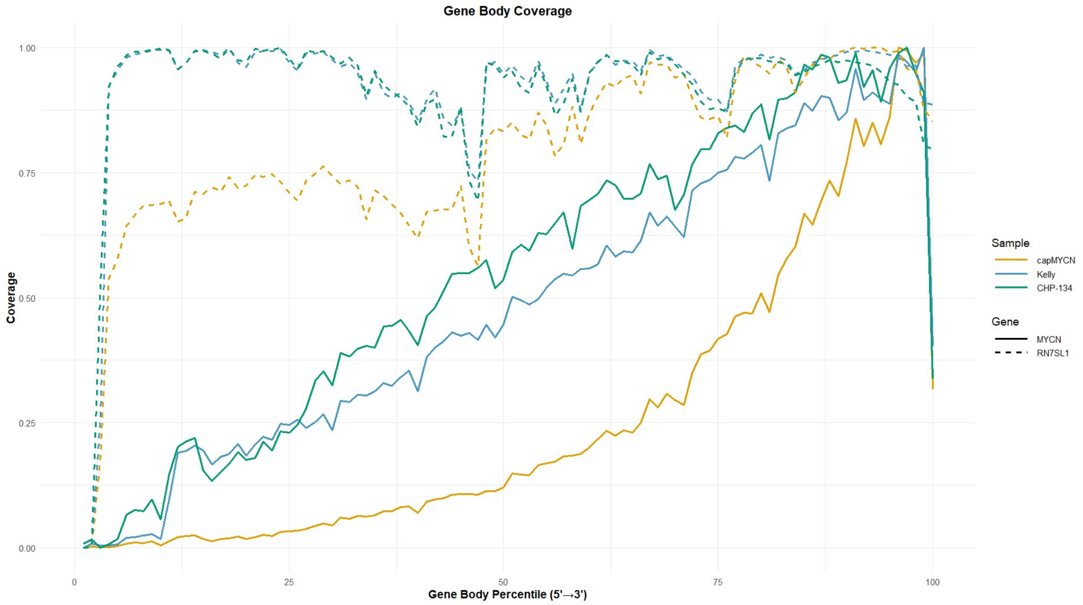

IGV Coverage plot:

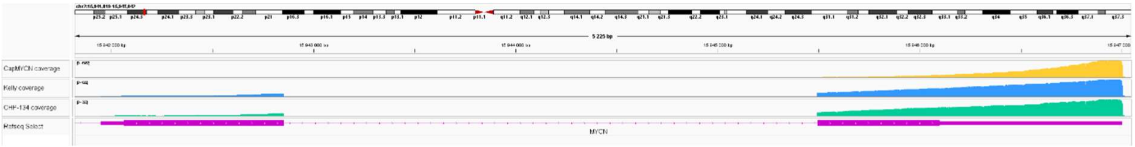

Conclusion; the reduced median read length, and sloped coverage curves from gene coverage plots and IGV coverage tracks, are consistent with significant gradual degradation of RNA during the enrichment protocol due to the prolonged handling of the RNA.

RNA degradation could be further minimized by incorporating RNase inhibitors during extraction and handling, and by using DEPC-treated RNase-free water and consumables.

Reference;

Wang, L., Wang, S., & Li, W. (2012). **RSeQC: quality control of RNA-seq experiments**. *Bioinformatics* (Oxford, England), 28(16), 2184–2185. http://doi.org/10.1093/bioinformatics/bts356
